# Relationship between risk factors and in-hospital mortality due to myocardial infarction by educational level: a national prospective study in Iran

**DOI:** 10.1186/s12939-014-0116-0

**Published:** 2014-11-27

**Authors:** Ali Ahmadi, Arsalan Khaledifar, Homeira Sajjadi, Hamid Soori

**Affiliations:** Department of Epidemiology and Biostatistics, School of Public Health, Shahrekord University of Medical Sciences, Shahrekord, Iran; Cardiology Department, School of Medicine, Hajar Hospital, Shahrekord University of Medical Sciences, Shahrekord, Iran; Social Determinants of Health Research Center, University of Social Welfare and Rehabilitation Sciences, Tehran, IR Iran; Safety Promotion and Injury Prevention Research center, Department of Epidemiology, School of Public Health, Shahid Beheshti University of Medical Sciences, 7th Floor, 2nd SBMU Headquarters Building, Parvaneh St., Velenjak Area, Chamran High Way, Tehran, Iran

**Keywords:** Myocardial infarction, Educational level, In-hospital mortality, Risk factor, Iran

## Abstract

**Introduction:**

Since no hospital-based, nationwide study has been yet conducted on the association between risk factors and in-hospital mortality due to myocardial infarction (MI) by educational level in Iran, the present study was conducted to investigate relationship between risk factors and in-hospital mortality due to MI by educational level.

**Methods:**

In this nationwide hospital-based, prospective analysis, follow-up duration was from definite diagnosis of MI to death. The cohort of the patients was defined in view of the date at diagnosis, hospitalization and the date at discharge (recovery or in-hospital death due to MI). 20750 patients hospitalized for newly diagnosed MI between April, 2012 and March, 2013 comprised sample size. Totally, 2511 deaths due to MI were obtained. The data on education level (four-level) were collected based on years of schooling. To determine in-hospital mortality rate and the associated factors with mortality, seven statistical models were developed using Cox proportional hazards models.

**Results:**

Of the studied patients, 9611 (6.1%) had no education. in-hospital mortality rate was 8.36 (95% CI: 7.81-8.9) in women and 6.12 (95% CI: 5.83-6.43) in men per 100 person-years. This rate was 5.56 in under 65-year-old patients and 8.37 in over 65-year-old patients. This rate in the patients with no, primary, high school, and academic education was respectively 8.11, 6.11, 4.85 and 5.81 per 100 person-years. Being woman, chest pain prior to arriving in hospital, lack of thrombolytic therapy, right bundle branch block, ventricular tachycardia, smoking and ST-segment elevation myocardial infarction were significantly associated with increased hazard ratio (HR) of death. The adjusted HR of mortality was 1.27 (95% CI: 1.06-1.52), 0.93 (95% CI: 0.77-1.13), 0.72 (95% CI: 0.57-0.91) and 0.82 (95% CI: 0.66-1.01) in the patients with respectively illiterate, primary, secondary and high school education compared to academic education.

**Conclusion:**

A disparity was noted in post-MI mortality incidence in different educational levels in Iran. HR of death was higher in illiterate patients than in the patients with academic education. Identifying disparities per educational level could contribute to detecting the individuals at high risk, health promotion and care improvement by relevant planning and interventions in clinics and communities.

## Introduction

Myocardial infarction (MI) is one of the most important health challenges in western and non-western communities, including Iran [1,2]. Despite promotion and usage of new therapeutic approaches, the morbidity and mortality relevant to MI are on rise in Iran and other Asian countries [3,4].

In view of the findings in different studies, the decreasing and/or increasing trend of MI and the associated mortality is not equally distributed in all population groups [5-7]. Relationship between health and socioeconomic status (SES) was reported in various works [8-10]. This correlation was also noted between SES, such as educational level (EL), income, occupation, and MI incidence and the associated mortality [11,12].

In a meta-analysis, a strong association was reported between MI incidence and low SES. An overall increased risk of MI among in low SES was found for all three indicators; income (relative risk [RR] = 1.71, 95% CI: 1.43 - 2.05), occupation (RR = 1.35, 95% CI: 1.19 -1.53) and education (RR = 1.34, 95% CI: 1.22 - 1.47).

The strongest associations were seen in high-income countries, such as the USA and Canada, and Europe, while the results were inconsistent or vague for middle and low-income countries [13]. Inconsistent findings seem to be attributable to the selection bias, nonresponsive, self-selection bias, information bias and limited number of prospective studies [14,15]. Other reasons for this inconsistency could be the difference in MI incidence rate, various genetic, lifestyle, and environmental factors or disparity of the disease risk factors in different SES. This issue requires further investigation in subsequent works.

Education has been hired as a proxy for SES in 30 studies in the Netherlands, Italy, Switzerland, Estonia, Finland, Denmark, France, Australia, Spain, Belgium, Lithuania, and Spain [13,16]. In Germany, the best index for assessing SES and disparity in health is education [17]. Validity and reliability of the above indices seem to be different in various communities. Education affects health both directly and indirectly. This index could be assessed for all individuals of the community. This measure is a strong predictor of health outcomes. In Iran, the economic data are not usually reliable, as with many other countries worldwide. The individuals could have more than one occupation and various income sources at the same time and be unwilling to mention their actual income [18-20]. Investigation of the relationship between MI incidence and in-hospital mortality determinants and identifying disparities per educational level could contribute to detecting the individuals at high risk, health promotion and care improvement by relevant planning and interventions in clinics and communities. Since no hospital-based, nationwide study has been yet conducted on the association between risk factors and in-hospital mortality due to myocardial infarction by educational level in Iran, the present study was conducted to study relationship between risk factors and in-hospital mortality due to MI by educational level.

## Methods

This is a prospective, hospital-based study in national scale in Iran. In this study, the data of 20750 MI cases registered in *Iranian Myocardial Infarction Registry* (*IMIR*) were used. *IMIR* collects the data from cardiac care units (CCUs) of all hospitals across the country [21]. All participants were patients hospitalized for newly diagnosed MI at 540 hospitals between April, 2012 and March, 2013. MI diagnosis was made using the criteria of the *International Classification of Diseases*-10th version (the code: I21) by the cardiologist [22]. The assessment of risk factors including blood pressure, diabetes, MI complications, place of MI, and the type of MI treatment was done based on the protocol codified by World Health Organization and Iran Ministry of Health and Medical Education per the information in the patients’ medical records (blood pressure of higher than 140/90 mmHg and fasting blood sugar of higher than 126 mg/dl were defined as respectively hypertension and type 2 diabetes). Years of schooling were categorized as zero year (no education or illiterate); 1–5 years (the first level or primary); 6–9 years (the second level or guidance); 10–12 years (the third level or high school); and more than 12 years (fourth level or academic), together used as a 4-mode variable for educational level.

The chi-square test was used to test for differences in frequencies, and student’s t-test and analysis of variance (ANOVA) were used to test for differences in continuous variables between groups. In-hospital mortality rate in the patients was calculated in person-year in the cohort of the patients defined by the date at diagnosis, hospitalization duration, and the date at discharge (recovery or death due to MI in hospital). In-hospital case fatality rate was calculated by the number of in-hospital MI death divided by number of reported hospitalizations due to registered MIs. For modeling and determining the factors relevant to in-hospital mortality per education, Cox proportional hazard models were used. In this prospective analysis, study follow-up was from definite diagnosis of MI to death. Totally, 3724983 person-years follow-ups were undertaken for 2511 deaths. The presuppositions of Cox model were checked and, except lack of thrombolytic therapy and ventricular tachycardia, were confirmed using Schoenfeld residuals (Ph test).

Firstly, all patients were classified by education and Cox analysis was run for each level of education. Then, Cox analysis was repeated for all patients with inclusion of different variables, such as educational level. Hazard ratio of mortality was calculated as crude in each educational level in zero model. Entering different variables, adjusting potential confounding variables in seven-mode models, and considering academic education as reference, we calculated the death HR in the patients by education as follows:

The first model was developed by age (quantitative) and gender; the second model by combination of the first model and its interaction with gender and EL; the third model by combination of the second model and history of the risk factors consisting of smoking, hypertension, diabetes, and blood lipid disorder; the fourth model by combination of the third model and therapeutic regimen such as thrombolytic therapy, percutaneous coronary intervention (PCI), and coronary artery bypass grafting (CABG); the fifth model by combination of the fourth model and ischemic pain pattern; the sixth model by combination of the fifth model and MI complications such as cardiac blocks, like left bundle branch block (LBBB), right bundle branch block (RBBB), ventricular fibrillation (VF), ventricular tachycardia (VT), and atrial fibrillation (AF); and the seventh model by combination of the sixth model and place and type of MI (anterior, posterior, inferior, lateral, ST-segment elevation myocardial infarction [STEMI], and non STEMI).

Myocardial infarctions are generally classified into STEMI and non STEMI. Electrocardiogram (ECG) testing was used to differentiate between two types of MI based on the shape of the tracing. For a person to qualify as having an STEMI, the ECG must show new ST elevation in two or more adjacent ECG leads. Considering seven-mode models, we developed the final model of patients’ mortality determinants with inclusion of education and other risk factors. To report in-hospital mortality rate, the date of birth was defined as an origin variable. To control the effect of age as a confounding variable, date of birth was used as the origin of time in Cox models and hence age was not reported in model. All analyses were done by Stata 12 Software.

## Results

Of 20750 studied patients, 9611 (46.32%) had no, 4941 (23.81%) primary, 1940 (9.35%) guidance, 2992 (14.42%) high school, and 1266 (6.1%) academic education. The mean age, hospitalization duration, the prevalence of risk factors like hypertension, diabetes, smoking, place and type of MI, complications, and type of treatment are shown in Tables 1 and 2 for educational levels. The in-hospital mortality rate due to MI was 8.36 (95% CI: 7.81-8.9) in women and 6.12 (95% CI: 5.83-6.43) in men per 100 person-years. This rate was 5.56 (95% CI: 5.24-5.87) in under 65-year-old patients and 8.37 (95% CI: 7.93-8.84) in over 65-year-old patients per 100 person-years. In-hospital mortality in the patients with no, primary, high school, and academic education was respectively 8.11, 6.11, 4.85, and 5.81 per 100 person-years respectively. The highest In-hospital mortality (15.37) was obtained in the women under 65 years with academic EL followed by the women above 65 years academic EL (13.04) and illiterate women above 65 years (10.66). The In-hospital mortality with 95% CI is shown in Table 3 for gender, age, and education. In-hospital case fatality rate in total patients and the patients with no, primary, high school, and academic education was respectively 12.1%, 14.7%, 11%, 8.4% and 11.3%. The crude HR of mortality was 1.41, 1.05, 0.78 and 0.83 in the patients with respectively no, primary, secondary and high school education compared to those with academic education. Controlling confounding variables, we illustrated the above ratios with 95% CI for seven-mode models in Table 4.

**Table 1:** **Demographic and medical characteristics in MI patients by educational levels in Iran, 2012-2013**

**Characteristic**	**Total n = 20750**	**Illiterate n = 9611**	**Primary n = 4941**	**Secondary n = 4932**	**University n = 1266**	**P-Value**
**Age (year)** *	61.2 ± 13.4	67.7 ± 12	58.7 ± 11.7	52.9 ± 11.2	52.9 ± 11	0.001
**Hospital stay (day)** *	6.5 ± 14.6	6.6 ± 14.6	6.5 ± 14.6	6.3 ± 14.1	7.04 ± 15.6	0.437
**Gender** **						
Men	15033 (72.4)	5488 (26.4)	3906 (18.8)	4426 (21.3)	1213 (5.8)	0.001
Women	5717 (27.6)	4123 (19.8)	1035 (4.9)	506 (2.4)	53 (0.26)	
**Smoking** **	5443 (26.2)	2313 (11.1)	1408 (6.7)	1385 (6.6)	337 (1.6)	0.001
**Hypertension** **	7376 (35.5)	4066 (19.6)	1584 (7.6)	1372 (6.6)	354 (1.7)	0.001
**Diabetes mellitus** **	4612 (22.2)	2243 (10.8)	1144 (5.5)	983 (4.7)	242 (1.1)	0.001
**Hyperlipidaemia** **	3710 (17.8)	1726 (8.3)	854 (4.1)	910 (4.4)	220 (1)	0.467

*Mean ± SD, **Frequency and percentage.

**Table 2:** **Clinical characteristics in MI patients by educational levels in Iran, 2012–2013**

**Characteristic**	**Total**	**Illiterate**	**Primary**	**Secondary**	**University**	**P-Value**
VF	511 (2.5)*	232 (1.1)	116 (0.56)	132 (0.64)	31 (0.15)	0.728
VT	1198 (5.8)	508 (2.4)	275 (1.3)	328 (1.5)	87 (0.42)	0.002
Lateral MI	990 (4.8)	494 (2.3)	223 (1.0)	222 (1.0)	51 (0.25)	0.115
Anterior MI	4332 (20.9)	2013 (9.7)	1042 (5.0)	991 (4.7)	286 (1.3)	0.236
Inferior MI	7179 (34.6)	3094 (14.9)	1711 (8.2)	1882 (9.0)	492 (2.4)	0.001
Posterior MI	853 (4.2)	383 (1.8)	233 (1.2)	192 (0.93)	45 (0.22)	0.087
STEMI	15729 (75.8)	6988 (44.4)	3842 (24.4)	3882 (24.6)	1017 (6.4)	0.001
Non-STEMI	5021 (24.2)	2623 (52.2)	1099 (21.8)	1050 (20.9)	249 (4.9)	0.001
PCI	1431 (6.9)	343 (1.6)	372 (1.7)	549 (2.6)	167 (0.80)	0.001
CABG	539 (2.6)	244 (1.1)	122 (0.59)	127 (0.61)	46 (0.22)	0.12
Lack of thrombolytic therapy	9222 (44.5)	3903 (18.8)	2209 (10.6)	2481 (11.9)	629 (3.0)	0.001
Chest pain	2229 (10.7)	1000 (4.8)	633 (3.0)	446 (2.1)	150 (0.72)	0.001

^*^Frequency and (%).

**VF-** ventricular fibrillation; **VT-** ventricular tachycardia; **MI**-myocardial infarction; **STEMI-** ST-segment elevation myocardial infarction; **NSTEMI**- non ST-segment elevation myocardial infarction; **PCI-** Percutaneous Coronary Intervention;** CABG**- Coronary Artery Bypass Grafting.

**Table 3:** **MI cases, CFR** ^**1**^ **and IMR** ^**2**^ **for gender, age, and education in Iran, 2012-2013**

**Gender**	**Age group**	**Education**	**Number (CFR %)**	**IMR: 95% CI** ^**3**^
**Women**	<65 years n = 2690	Illiterate	1533(56.9)	7.09:6.13-8.19
		Primary	705(26.2)	5.8:4.57-7.36
		Secondary	406(15.1)	4.96:3.53-6.79
		University	46(1.7)	15.37:7.69-30.7
		Total women <65	2690(47)	6.49:5.7 -7.2
	≥65 years n = 3027	Illiterate	2590(85.5)	10.66:9.78-11.62
		Primary	330(10.9)	5.58:4.02-7.74
		Secondary	100(3.3)	3.49:1.65 – 7.73
		University	7(0.23)	13.04:1.83 – 19.6
		Total women ≥65	3027(53)	9.8:9–10.6
	Total women		5717(27.6)	8.36:7.8 – 8.9
**Men**	<65 years n = 9814	Illiterate	2197(22.3)	5.68:4.98 – 6.48
		Primary	2767(28.2)	5.74:5.1 – 6.45
		Secondary	3800(38.7)	4.56:4.06 – 5.11
		University	1050(10.6)	5.88:4.89 -7.06
		Total men <65	9814(65.3)	5.3:4.9 -5.6
	≥65 years n = 5219	Illiterate	3291(63)	8.07:7.39 – 8.81
		Primary	1139(21.8)	7.24:6.2 – 8.46
		Secondary	626(12)	6.57:5.28 -8.18
		University	163(3.2)	4.29:2.73 -6.72
		Total men ≥65	5219(34.7)	7.5:7 – 8.1
		Total men	15033(72.4)	6.12: 5.8 -6.43
**Total**		Illiterate	9611(46.3)	8.11:7.7 – 8.5
		Primary	4941(23.8)	6.11: 5.6 – 6.6
		Secondary	4932(23.8)	4.85:4.4 – 5.3
		University	1266(6.1)	5.81: 4.9 – 6.8

^1^Case fatality rate; ^2^In-hospital mortality rate (per 100 person-years); ^3^Confidence interval.

**Table 4:** **Modeling of HR (CI 95%)** ^*^ **for MI mortality in educational levels in Iran, 2012-2013**

**Models**		**Illiterate**	**Primary level**	**Secondary**
0	Unadjusted HR	1.4: 1.18-1.67 P < 0.001	1.05:0.87-1.27 P = 0.557	0.81:0.67-0.98 P = 0.037
1	Adjusted for age and gender	1.11:0.93-1.34 P = 0.223	0.96: 0.8-1.16 P = 0.716	0.8:0.66-0.97 P = 0.025
2	Above + interaction of gender with EL	1.7: 1.09-2.65 P = 0.019	1.31:0.82-2.07 P = 0.249	1.19: 0.73-1.94 P = 0.47
3	Above + past medical history	1.69:1.08-2.63 P = 0.021	1.28:0.81-2.04 P = 0.280	1.18:0.72-1.93 P = 0.49
4	Above + treatment regime	1.72:1.1-2.7 P = 0.017	1.32:0.83-2.1 P = 0.237	1.27:0.78-2.08 P = 0.329
5	Above + Ischemic Pattern Pain	1.05:0.87-1.26 P = 0.56	0.86:0.72-1.04 P = 0.143	0.79:0.66-0.96 P = 0.022
6	Above + complication of MI	1.04:0.87-1.25 P = 0.629	0.85:0.71-1.03 P = 0.114	0.78: 0.64-0.95 P = 0.014
7	Above + MI Type	1.05: 0.87-1.26 P = 0.575	0.95:0.75-1.05 P = 0.306	0.78: 0.65-0.95 P = 0.015

^*^Hazard ratio (confidence interval 95%).

Model2: age, gender and interaction of gender with EL. Model3: smoking + Type 2 Diabetes Mellitus + Hypertension + Hyperlipidaemia. Model4: PCI + CABG + Thrombolytic, model5: Chest Pain + pain left arm + dyspnea + sweating + vomiting + nausea + jaw pain. Model6: RBBB, LBBB, AF, VF, VT, Model7: STEMI + non-STEMI, MI Status.

The adjusted HR of mortality was 1.27, 0.93, 0.72, and 0.82 in the patients with respectively no, primary, secondary, and high school education compared to those with academic education in the multivariate analysis. The significant determinants in the final Cox model of the patients’ mortality are shown in Table 5 for total patients. The HR of mortality for diabetes mellitus in univariate analysis was significant (HR: 1.1, 95% CI: 1–1.2, P = 0.039), but in multivariate analysis, it was not significant (HR: 1.06, 95% CI: 0.97 – 1.17, P = 0.172).

**Table 5:** The hazard ratio of factors associated with MI mortality in patients in Iran (2012–2013)

**Factor**	**Univariate analysis**		**Multivariate cox model**	
	**HR:CI95%** *	**P-Value**	**HR:CI95%**	**P-Value**
**Gender**				
**Men**	Ref.	-	-	-
**women**	1.38:1.27-1.5	0.001	1.28:1.17-1.40	0.001
**Education**				
Illiterate	1.41:1.18-1.67	0.001	1.27:1.06-1.52	0.007
Primary	1.05:0.87-1.27	0.557	0.93:0.77-1.13	0.503
Secondary	0.78:0.62-0.99	0.041	0.72:0.57-0.91	0.006
High school	0.83:0.67-1.02	0.083	0.82:0.66-1.01	0.06
University	Ref.	-	-	-
**Diabetes mellitus** *	1.1:1–1.2	0.039	1.06:0.97-1.17	0.172
**Smoker** *	1.31:1.2-1.42	0.001	1.16:1.06-1.27	0.001
**VT** *	2.19:1.93 – 2.49	0.001	1.67:1.46-1.90	0.001
**STEMI** *	3.27: 3.01 -3.55	0.001	1.32:1.18-1.48	0.001
**RBBB** *	2.81:2.23 – 3.55	0.001	2.45:1.94-3.11	0.001
**Chest pain** *	4.68:4.3-5.08	0.001	4.06:3.73-4.43	0.001
**Lack of TT** *	1.91:1.77-2.07	0.001	1.57:1.44-1.72	0.001
**PCI** *	0.42:0.34 – 0.53	0.001	0.61:0.49-0.77	0.001

^*^Hazard ratio (confidence interval 95%).

**VT-** ventricular tachycardia; **STEMI-** ST-segment elevation myocardial infarction; **RBBB-** right bundle branch block;** TT**- thrombolytic therapy; **PCI-** percutaneous coronary intervention; *The variables were entered as dichotomous (0 & 1) and 0 was set as reference.

## Discussion

In this study, in-hospital mortality rate in women and men was respectively 8.36 and 6.12 in 100 person-years. This rate was 8.11 in the patients with no education and 5.81 in the patients with academic education. The mortality rate was higher in women than men and in the patients over 65 years than those under 65 years. The notable points in our study were avoidance of biases of selection and information and conduction of a hospital-based, large study with the findings generalizable to the whole country. Cox analysis and modeling were other advantages of our work in comparison to the works conducted in similar countries where other analyses have been used due to the defined MI cohort.

In China, low education was associated with increased MI risk (OR = 1.45) [6]. In our study, illiteracy was associated with increased death HR due to MI (HR = 1.27) and the associated factors with death in illiterate patients were different from those in the patients with other educational levels. However, presence of main cardiovascular disease risk factors in the illiterate individuals indicates that disparity in post-MI mortality could be largely prevented through offering pre-hospital diagnostic and therapeutic services and hospital care to illiterate patients. Also, our findings are not consistent with a study in Iran, reporting that smoking, age, and gender were not determinants of in-hospital mortality due to MI [7]. Small sample could explain this inconsistency between the findings. The outcome of that study was 65 cases of in-hospital mortality and 500 cases of survival while our study investigated 2511 cases of mortality. One of strengths of our study was measurement of education by four levels, while in the study of Germany this variable was measured by two levels, low and high [17]. The rarity or lack of illiteracy in Germany and difficulty with fitting the German educational system into the International Standard Classification of Education could explain this classification. But, illiteracy is unfortunately high in Iran especially in women. In Germany, 19.1% of the patients with poor education died (long-term mortality) compared with 13.1% with higher education; the corresponding rates of in-hospital case fatality rate and in-hospital mortality rate in the present study were obtained respectively 46.2% and 11.3 per 100 person-years in patients with poor education, and 19.17% and 5.81 per 100 person-years in those with high education, higher compared to the long-term mortality in the study of Germany. In the study of Germany after confounding variables were controlled, the education had no effect on mortality in the total sample and the participants under 65 years. Also, low education was associated with increased death (HR = 1.44) *only* in elderly patients. But, the education was an important variable and was associated with death HR in all ages in our study.

In Finland, the mortality rate was 5.21 in 35- to 64-year-old men and 11.31 in women [23]. Mortality rate in our study was higher than that obtained 30 days after MI in studies of Sweden and the UK with respectively 7.6 and 10.5 in 100 person-years. In Sweden and the UK, MI mortality was different for age, gender, blood pressure, diabetes, smoking, type of treatment, and type of MI, which is consistent with our study [24]. Although in our study, consistent with the study of Finland, death HR was higher in the patients with low SES and women, the mortality rate was higher in Iranian men compared to Finnish men and higher in Finnish women compared to Iranian women. Since the highest MI incidence worldwide has been reported in Finland, death HR seems to be higher in our study compared to the study of Finland. This requires further investigation. The in-hospital case fatality rate in our study was similar to that in Puerto Ricans, in whom the mortality rate in men and women was reported respectively 8.6% and 6% [25].

The results of our study are in agreement with a study that examined the association of education with MI in the USA, reporting an association between low education and increased risk of fatal and non-fatal MI [26].

Being women, chest pain prior to arriving in hospital, lack of thrombolytic therapy, RBBB, smoking and STEMI were significantly associated with increased death HR. These findings are not consistent with the studies reporting a lower decrease in type 2 diabetes, hypertension, and smoking in the individuals with high education compared to those with low education [27-29]. This observation could be explained by higher physical activity among the illiterate individuals who possibly live in non urban regions. The difference in MI incidence and the associated mortality has been also reported by other studies among age groups and between genders, in agreement with our results. MI incidence is higher in men than women. In some studies, this incidence is higher in women than men and in some others it is relatively equal. The difference in mortality between men and women is dependent on age and type of MI (STEMI). Besides, biological factors could be the reason for this difference. In women, the type of MI is mainly non STEMI. Decreasing disparity between genders could contribute to the decrease in MI-associated mortalities [30,31]. Smoking caused an increase in post-MI death HR in the patients while education was controlled [32,33]. Policy making and implementation of interventional programs in this group of patients could decrease the death risk. In view of the risk factors attributed to increased death risk in patients with different educational levels, the mortality due to MI (the most prevalent reason for death in Iran) could be decreased by implementing the programs adjusted to the mentioned risk factors at the community and case scale, such as offering special nursing services, and hence the concerns of health community professionals could be mitigated [34,35].

## Limitations

Failure to calculate mortality rate 28 days after MI incidence was a limitation of the present study. If this index is calculated, the possibility of comparing the findings with other studies’ will increase. This index is recommended to be measured in future studies of Iran. Failure to measure income and occupation, and low number of cases with academic education in women were other limitations of the present study.

## Conclusions

In this study, in-hospital mortality rate and case fatality rate due to MI were reported by age, gender, and education based on a hospital-based work in national scale in Iran. Also, in this study the factors relevant to the patients’ mortality were modeled and determined in different educational levels. Being women, chest pain prior to arriving in hospital, lack of thrombolytic therapy, RBBB, VT, smoking, and STEMI were significantly associated with increased death risk. A disparity was noted in post-MI mortality incidence in different educational levels in Iran. HR of death in illiterate patient was higher than in the patients with academic education. Identifying disparities per educational level could contribute to detecting the individuals at high risk, health promotion and care improvement by relevant planning and interventions in clinics and communities.

## Abbreviations

MI: Myocardial infarction
EL: Education level
SES: Socioeconomic status
IMIR: Iranian myocardial infarction registry
PCI: Percutaneous coronary intervention
CABG: Coronary artery bypass grafting
CI: 95% confidence intervals
NSTEMI: None ST-segment elevation myocardial infarction
STEMI: ST-segment elevation myocardial infarction
LBBB: Left bundle branch block
RBBB: Right bundle branch block
VF: Ventricular fibrillation
ECG: Electrocardiographic
VT: Ventricular tachycardia
AF: Atrial fibrillation
